# Effect of Exogenous Alpha-B Crystallin on the Structures and Functions of Trabecular Meshwork Cells

**DOI:** 10.1155/2018/7875318

**Published:** 2018-04-17

**Authors:** Hui Xu, Li Zhu, Yu Wang, Yongzhen Bao

**Affiliations:** Department of Ophthalmology, Peking University People's Hospital, Eye diseases and Optometry Institute, Beijing Key Laboratory of Diagnosis and Therapy of Retina and Choroid Diseases, College of Optometry, Peking University Health Science Center, Beijing, China

## Abstract

**Purpose:**

Secondary open-angle glaucoma may develop as a postoperative complication of early childhood cataract surgery. Its mechanism is poorly understood. Surgical removal of cataracts is typically incomplete, and we estimate that this disease is associated with alpha-B crystallin (CRYAB) secreted from the retained lens material. This study, for the first time, focused on the role of CRYAB in undesired changes of the structures and functions in trabecular meshwork (TM) cells.

**Methods:**

Cell proliferation and migration were assessed using a cell counting kit-8 (CCK8) and transwell assay analysis, respectively. Immunofluorescence (IF), quantitative real-time PCR (Rt-qPCR), and Western blot were performed to determine the effect of CRYAB on F-actin, tight junctions, and the expression of epithelial to mesenchymal transition- (EMT-) associated proteins in TM cells.

**Results:**

CRYAB promoted proliferation (*p* < 0.0001), migration (*p* < 0.001), and F-actin reorganization in TM cells. There were statistically significant increases in the mRNA and protein levels of zo-1, cadherin-N, and vimentin (all *p* < 0.0001) and cadherin-E decreased (*p* < 0.0001) and the mRNA level of claudin-1 increased (*p* < 0.0001) compared to those of the control group.

**Conclusion:**

All of the changes in structures and functions first observed in the TM cells after exposure to CRYAB resembled alterations seen in primary open-angle glaucoma, suggesting that CRYAB might be related to the pathogenesis of secondary open-angle glaucoma after congenital cataract surgery.

## 1. Introduction

Cataracts are a major cause of visual disability in childhood. For children with a significant disorder of visual acuity in infancy, early congenital cataract extraction combined with posterior capsule resection and preoperative vitrectomy is conducive for restoring vision and reducing amblyopia and blindness. However, in the first year after birth, lensectomy increases the incidence of postoperative secondary open-angle glaucoma [1–3], a slightly threatening complication with an incidence ranging from 6% to 58.7%, according to the variable population definition and follow-up time [4–9]. Once it occurs, treatment is difficult and prognosis is poor. The pathogenesis of this kind of glaucoma has been researched, and it has been associated with some suspected risk factors, consisting of a preexisting ocular abnormality, operation at early age, chronic postoperative inflammation, and retained lens material, and factors, including IL-4 and TGF-*β* in the aqueous humor of the eye [10, 11]. However, the mechanism responsible for secondary open-angle glaucoma is poorly understood to date. One study points out that the volume of trabecular cells (TM) increases as well the expression of genes and proteins related to cell morphological characteristics, inflammatory response, and ion balance regulation after TM are cocultured with lens epithelial cells (LECs), which is similar to the change in TM in primary open-angle glaucoma [12].

In addition, alpha-B crystallin (CRYAB) may play a role in the incidence of cataracts and AMD by protecting cells from apoptosis, regulating cell signals and resisting oxidative stress [13, 14]. Previous studies show that *α*B-crystallin promotes EMT in hepatocellular carcinoma cells [15] and plays an important role in pulmonary fibrosis [16]. Surgical removal of cataract is typically incomplete and exposes the internal surfaces of the equatorial and posterior lens capsule to the aqueous humor, which is covered with LECs secreting CRYAB into the aqueous humor. CRYAB likely causes the above changes in trabecular extracellular matrix and promotes epithelial to interstitial transformation, leading to the incidence of postoperative glaucoma. This prompted us to undertake this study, for the first time, to investigate whether CRYAB secreted from the retained lens material negatively affects the TM cellular structures and functions by EMT and its relation with the pathogenesis of secondary open-angle glaucoma.

## 2. Methods

### 2.1. Cell Culture

The human trabecular meshwork cell line was purchased from the American Type Culture Collection (ATCC) (Manassas, VA). The cells were maintained in T25 and were incubated with DMEM medium (Invitrogen, Carlsbad, CA) supplemented with 10% fetal bovine serum (FBS) (Invitrogen, USA), 100 units/ml penicillin, and 100 *μ*g/ml streptomycin (Invitrogen, USA) in a tissue culture incubator at 37°C with 5% CO_2_. Once they reached 80∼90% confluence, the TM cells were passaged (1 : 3∼1 : 4) every 2 or 3 days.

### 2.2. Cell Proliferation

We inoculated the cell suspension (100 *μ*l/well) in a 96-well plate at 37°C in 5% CO_2_ after adding CRYAB at 1 ng/ml, 10 ng/ml, and 100 ng/ml. Twenty-four hours later, we added 10 *μ*l of the CCK-8 solution to each well of the plate for one hour incubation and then measured the absorbance at 450 nm using a microplate reader.

### 2.3. Cell Migration

The transwells had an 8 *μ*m pore size and were 6.5 mm in diameter (Costar; cat. no. 3422). The cells (4 × 103) were placed in the upper chamber (Costar, Cambridge, MA) with a volume of 200 *μ*l of serum-free medium, and 10% FBS, with concentrations of crystallin (10 ng/ml), without the other test substances was placed in the bottom chamber in a volume of 600 *μ*l per well. The cells were fixed in 4% paraformaldehyde for 20 min, stained with DAPI for 10 min and washed in PBS. The nonmigrated cells in the upper chamber were gently removed using a cotton swab. Cell migration was counted by the number of cells that moved through the filter towards the lower surface in five random fields per filter under a microscope.

### 2.4. Cytoskeleton

The cells were fixed for 20 minutes in 4% paraformaldehyde (Sigma-Aldrich, St. Louis, MO) and were washed for 5 min in PBS 3 times. Then, they were permeabilized with 0.1% TRITON X-100 (Sigma-Aldrich) in PBS at room temperature and washed 3 times in PBS. The cells were stained with a 50 *μ*g/ml fluorescent phalloidin conjugate solution in PBS (containing 1% DMSO from the original stock solution) for 40 minutes at room temperature in the dark and were washed 3 times with PBS to remove unbound phalloidin conjugate. The nuclei of the TM cells were stained with a 4′, 6-diamidino-2-phenylisndole (DAPI) antibody (Sigma-Aldrich). For each experimental condition, three biological repeats were performed.

### 2.5. Immunofluorescence

The cells were fixed and permeabilized following the same procedure as cytoskeleton staining. Then, they were blocked with 1% BSA in PBS for 1 h and incubated with rabbit polyclonal primary antibodies against zo-1, claudin-1, cadherin-E, and cadherin-N (1 : 100 Abcam, England) and a mouse polyclonal primary antibody against vimentin (Abcam, England) overnight at 4°C. Secondary antibodies were used, including TRITC-conjugated goat anti-rabbit IgG and FITC-conjugated goat anti-mouse IgG (1 : 200 Abcam, England). They were incubated for 2 h at 37°C in a humidified atmosphere in the dark. The nuclei of the TM cells were stained with DAPI (1 : 1000; Sigma, USA). The samples were analyzed in a drop of PBS under a fluorescence microscope at this state.

### 2.6. RNA Extraction and Rt-qPCR

Total RNA was obtained from the TM cells cultured in 10 ng/ml CRYAB with a Total RNA kit (Omega, USA) and was reverse transcribed into cDNA (1 *μ*g of isolated total RNA) using a qPCR RT Kit (TOBOYO, Japan) according to the manufacturer's instructions. The RNA concentrations were quantified with a Nano instrument (NanoDrop Technologies, Wilmington, DE, USA). Rt-qPCR was carried out using a SYBR Green PCR Master Mix (TOYOBO, Japan). The system of each PCR reaction contained 4 *μ*l of cDNA, 0.8 *μ*l of forward and reverse primers (PCR primers were listed), and 10 *μ*l of SYBR Green PCR Master Mix, with a final volume of 20 *μ*l. All the PCR amplification reactions were performed on a MiniOpticon qPCR (Bio-Rad, USA). The specific primers were the following: zo-1 (forward primer (F), 5′-ACCAGTAAGTCGTCCTGATCC-3′ and reverse primer (R), 5′-TCGGCCAA ATCTTCTCACTCC-3′); claudin-1 (F, 5′-CGAGAGCTACAC GTTCACGG-3′ and R, 5′-GGGTGTCGAGG GAAAAA TAGG-3′); cadherin-E (F, 5′-AAAGG CCCATTTCCTAAAAACCT-3′ and R, 5′-TGCGTTCTCTATCCAGAGGCT-3′); cadherin-N (F, 5′-AGCCAACCTTAAC TGAGGAGT-3′ and R, 5′-GGCAAGTT GATTGGAG GGATG-3′); and vimentin (F, 5′-GACGCCATCAACACCGAGTT-3′ and R, 5′-CTTTGTCGTTGGTTAG CTGGT-3′). The mRNA expression was normalized to the endogenous reference gene GAPDH (F, 5′-TGGAGAAAAT CTGGCACCAC-3′ and R, 5′-ACCACCCTGTTGCTGTAGCCA A-3′) and was analyzed using the 2^−ΔΔCt^ comparative threshold method.

### 2.7. Protein Extraction and Western Blotting

Total protein from the TM cells was extracted using a RIPA lysis buffer (Thermo, USA). The protein concentration was detected by a bicinchoninic acid assay kit (Beyotime, China). Equal amounts of total protein (30 *μ*g) were separated by 10% SDS-polyacrylamide gel electrophoresis and were transferred to polyvinylidene difluoride membranes (Millipore, Bedford, MA, USA) and were blocked with 5% nonfat skim milk in Tris-buffered saline supplemented with Tween 20 (TBST) for 1.5 h. The proteins were incubated with rabbit polyclonal primary antibodies against zo-1, cadherin-E, cadherin-N, vimentin, and GAPDH (1 : 1000, Cell Science Tech, USA) overnight at 4°C and were then washed and incubated in a secondary antibody conjugated to horseradish peroxidase (1 : 2000, Cell Science Tech, USA) for 1 h at room temperature. The bands were analyzed using ImageJ software (http://imagej.nih.gov/ij/; provided in the public domain by the National Institutes of Health, Bethesda, MD, USA). GAPDH was used as the loading control.

### 2.8. Statistical Analysis

All the data were analyzed with the software package SPSS19.0 (SPSS Inc., Chicago, IL, USA). The statistical analysis was performed using one-way analysis of variance and a Student's *t*-test. *p* < 0.01 was considered statistically significant. The data are expressed as the means ± standard deviation (SD).

## 3. Result

### 3.1. Cell Proliferation

**Figure 1:**
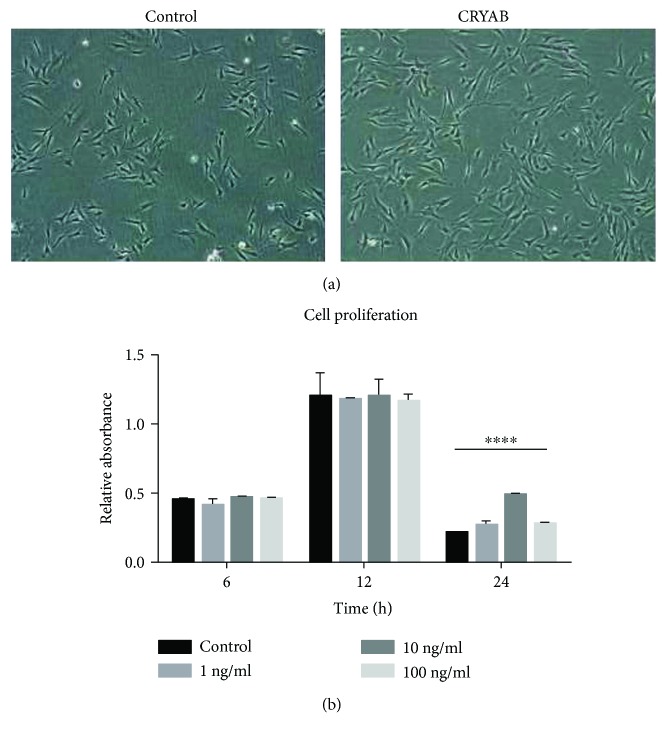
(a) Light micrographs of the TM cells treated with CRYAB (TM cells grown alone served as the control, 100x magnification). (b) CRYAB induced proliferation in TM cells. TM cell proliferation was determined with CCK8 after 6 h, 12 h, and 24 h incubation with 1 ng/ml, 10 ng/ml, and 100 ng/ml CRYAB. The 10 ng/ml CRYAB for 24 h treatment increased TM cell proliferation. ^∗∗∗∗^*p* < 0.0001 versus the untreated control.

In the current study, we first treated the TM cells with a series of concentrations of CRYAB to induce proliferation. The protein concentration ranged from 1 to 10 ng/ml, and the total treatment time was 6 h, 12 h, and 24 h. A cell counting kit-8 was then used to assess cell proliferation by a microplate reader. At one of the tested concentrations of 10 ng/ml, CRYAB induced significant proliferation in the TM cells compared to the control condition of 1 ng/ml (Figure 1). We then chose 10 ng/ml as the protein concentration to be used in the rest of the study, as it induced a high percentage of proliferation.

### 3.2. Cell Migration

**Figure 2:**
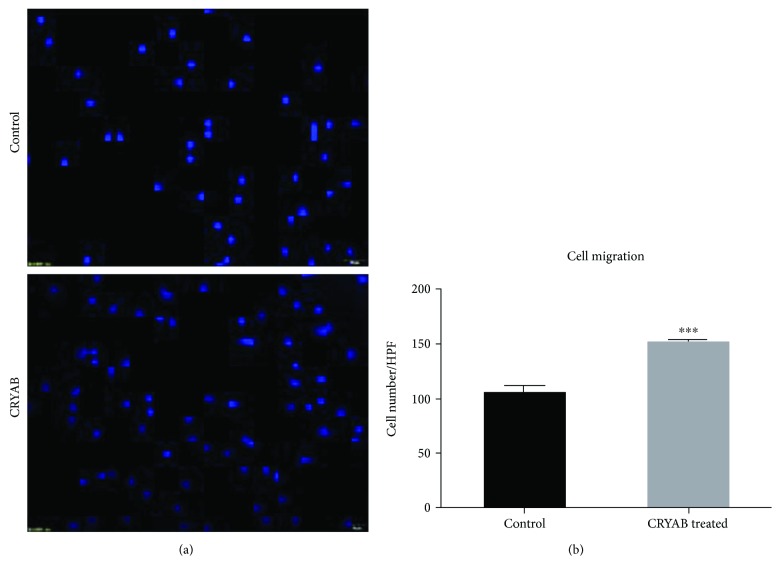
(a, b) CRYAB induced migration in TM cells. TM cell migration in response to CRYAB treatment was measured using a transwell assay (100x magnification). The amounts were assessed by the mean number of migrated cells. The number of migrated cells per high-power field (HPF) was shown. ^∗∗∗^*p* < 0.001 versus the untreated control.

In the cell migration assay, the TM cells were measured in a modified Boyden chamber, in which the TM cells migrated through a porous membrane. The mean number of migrated cells in the CRYAB-treated TM cells (10 ng/ml) was significantly higher than the mean number of migrated control cells (Figures 2(a) and 2(b)).

### 3.3. Cytoskeleton Reorganization

**Figure 3:**
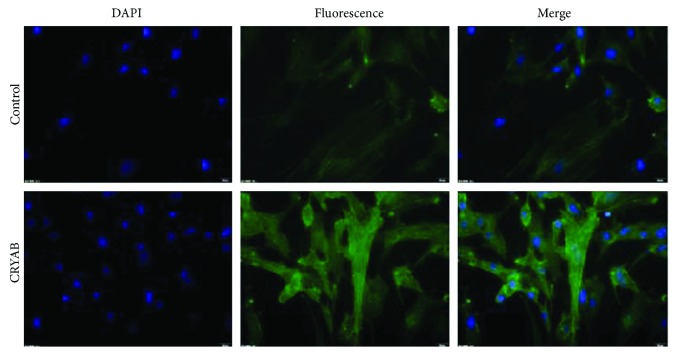
Immunofluorescence after incubation with CRYAB for 24 hours showed that F-actin protein in the CRYAB-treated TM cells irregularly accumulated inside the cell membrane.

The cell IF showed that F-actin in CRYAB-treated TM cells irregularly accumulated inside the cytoplasm (Figure 3).

### 3.4. Effect of CRYAB on Tight Junction Protein Expression

**Figure 4:**
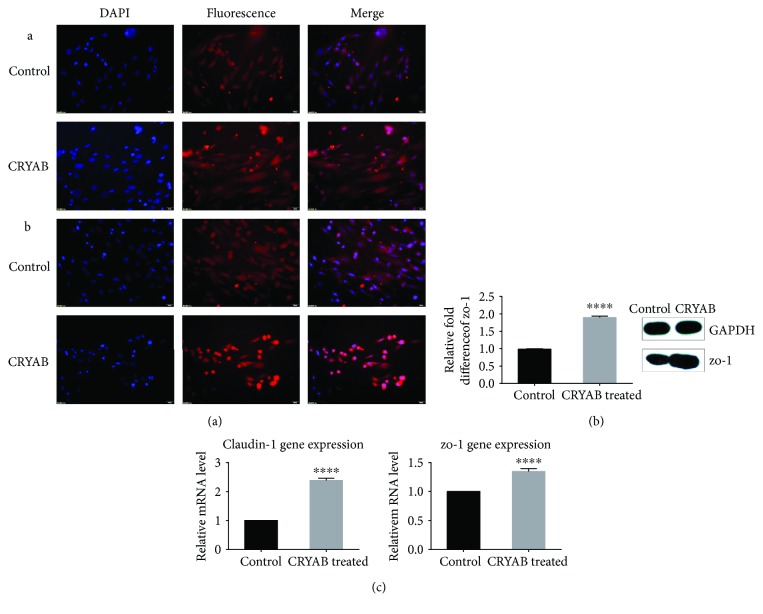
Effect of CRYAB on the expression of zo-1 and claudin-1 in TM cells. (a) Immunofluorescence manifestation of zo-1 and claudin-1. Immunofluorescence verified the upregulation of tight junction proteins (A: zo-1; B: claudin-1). (b) Western blot analysis of zo-1 expression. TM cells were exposed to 10 ng/ml CRYAB for 24 h. The expression of zo-1 increased under CRYAB conditions compared with the control. (c) Rt-qPCR analysis of zo-1 and claudin-1 mRNA expression. Compared with that of the control, the expression of zo-1 and claudin-1 was upregulated in response to CRYAB. The data represent the mean ± SD of three independent experiments. ^∗∗∗∗^*p* < 0.0001 versus the control.

Meanwhile, the level of zo-1, with the application of CRYAB, was significantly upregulated (Figure 4(a), A). Western blot indicated results that were consistent with the IF data, showing that zo-1 was significantly regulated in the CRYAB-treated TM cells (Figure 4(b), *p* < 0.0001), and there were significant differences in the zo-1 and claudin-1 mRNA levels (Figure 4(c), *p* < 0.0001).

### 3.5. Effect of CRYAB on EMT

**Figure 5:**
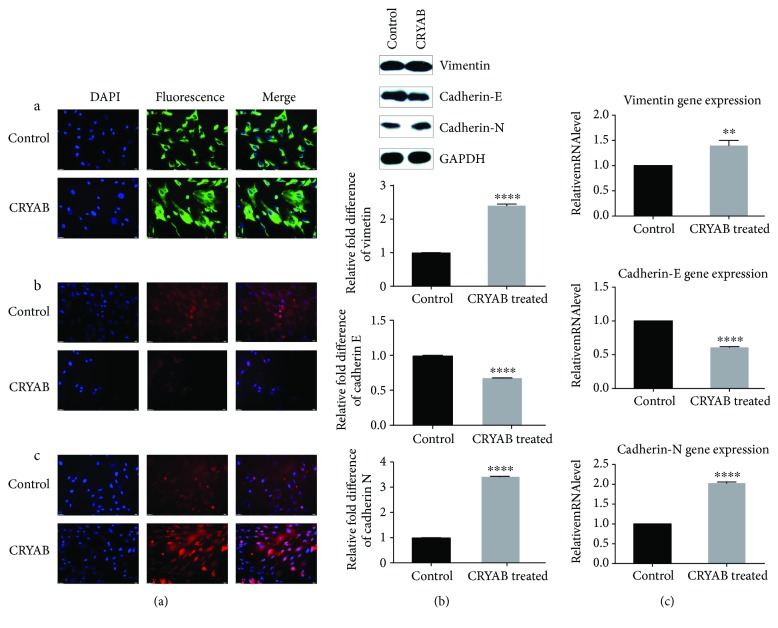
Effect of CRYAB on EMT in TM cells. (a) Immunofluorescence manifestation of vimentin, cadherin-E, and cadherin-N. Immunofluorescence verified the upregulation of vimentin and cadherin-N and the downregulation of cadherin-E (A: vimentin; B: cadherin-E; C: cadherin-N). (b) Western blot analysis of vimentin, cadherin-E, and cadherin-N expression. TM cells were exposed to 10 ng/ml CRYAB for 24 h. The expression of vimentin and cadherin-N increased, whereas cadherin-E decreased under CRYAB conditions compared with the control. (c) Real-time PCR analysis of vimentin, cadherin-E, and cadherin-N mRNA expression. Compared with the control, the expression of vimentin and cadherin-N was upregulated, while cadherin-E was downregulated in response to CRYAB. The data shown represent the mean ± SD of three independent experiments. ^∗∗^*p* < 0.01 and ^∗∗∗∗^*p* < 0.0001 versus the control.

We also determined how CRYAB affected the levels of proteins related to EMT in TM. We detected traces of cadherin-N and vimentin, as shown in Figures 5(b) and 5(c), and the levels of cadherin-N and vimentin were upregulated. In contrast, cadherin-E was downregulated (all *p* < 0.0001). Additionally, the expression of cadherin-E was specifically weakened, while vimentin and cadherin-N appeared with more intense staining compared with the control group (Figures 5(a), A; 5(b); and 5(c)).

## 4. Discussion

The experiments presented here first show that normal TM cells resulted in changes in their function as well as in their protein and gene expression by EMT. Many of the changes resembled alternations seen in ocular tissues of patients with primary open-angle glaucoma [17–19].

We reported that CRYAB promoted the proliferation and migration of TM cells. In addition, the proliferation and migration of TM cells are associated with glaucoma. Glaucoma develops because of an imbalance between aqueous production and its outflow through the trabecular meshwork. We found that when TM cells were incubated with a variety of concentrations of CRYAB, cell proliferation and migration were significantly increased at concentrations of 10 ng/ml. Excessive proliferation and migration may obstruct drainage angle tissue accompanied by the increase of intraocular pressure (IOP). Changes in the cytoskeleton of TM cells are well studied [20–22]. The cytoskeleton is involved with cell volume, shape, and adhesion to the extracellular matrix and to neighboring cells. CRYAB-treated TM cells present alternations in the F-actin, as we observed a major reorganization of the microfilament structure.

Tight junctions, or zo-1 and claudin-1, form a continuous barrier of fluids across the epithelium, which functions in the regulation of paracellular permeability and the maintenance of cell polarity [23]. The overexpression of tight junction proteins induces cell interaction damage as observed in glaucoma [24, 25]. Our study showed that CRYAB upregulated zo-1 at both the protein and mRNA levels, which might impair aqueous outflow by changing the connections between TM cells. However, we did not observed a change that was consistent with the mRNA in claudin-1 by means of Western blot; thus, we determined that TM cells have a post transcriptional modification process that needs to be further researched.

CRYAB seemed to trigger the expression of genes associated with EMT, then resulted in cell migration. The damage to the trabecular cells is mainly reflected in two aspects, including EMT and the deposition of extracellular matrix (ECM). EMT is mainly reflected by the gradual loss of epithelial properties. E-cadherin expression is downregulated, while mesenchymal phenotypes, such as vimentin, fibronectin, and cadherin-N expression, are upregulated [26]. We observed that TM cells treated with CRYAB had upregulated N-cadherin in addition to the loss of E-cadherin, which indicated that the TM cells acquired mesenchymal properties and displayed reduced intracellular adhesion, because E-cadherin is considered an active suppressor of invasion [27–29]. Vimentin is an intermediate filament of mesenchymal origin, which has conventional expression in TM, whereas its overexpression, as in our study, may cause TM cells to lose their epithelial properties. The acquiring of mesenchymal properties prompted the migration of the TM cells, as we observed.

Previous reports present evidence of TGF-2 in the pathogenesis of POAG [30, 31]. Moreover, TGF-2, which affects EMT, is elevated in the aqueous of patients with glaucoma [32–34]. There is convincing evidence that TGF-2 upregulates CRYAB and that the upregulation of CRYAB occurs with the up/downregulation of EMT markers [35]. In addition, a recent study suggests a direct interaction of CRYAB with beta-catenin [36], which raises the possibility of the role of CRYAB in EMT. These data suggest that CRYAB might be a target to block the secondary open-angle glaucoma. The control of inflammation after surgery provides benefits to it as well.

Overall, all the changes that the TM cells experienced after the exposure to CRYAB resembled alterations seen in primary open-angle glaucoma, which implied that CRYAB affected the TM cellular structures and functions by EMT and might be responsible for the development of secondary open-angle glaucoma. In future research, the specific mechanism should be further studied.
